# Cost‐Outcome of Radiotherapy for Local Control and Overall Survival Outcomes for Selected Cancers

**DOI:** 10.1111/1754-9485.70000

**Published:** 2025-08-05

**Authors:** Jesmin Shafiq, Vikneswary Batumalai, Karen Wong, Nasreen Kaadan, Alexandra Powell, Geoff P. Delaney, Shalini K. Vinod

**Affiliations:** ^1^ Collaboration for Cancer Outcomes Research and Evaluation (CCORE) Ingham Institute for Applied Medical Research Liverpool New South Wales USA; ^2^ Cancer Therapy Centre Liverpool Hospital, South Western Sydney Local Health District Sydney New South Wales Australia; ^3^ School of Clinical Medicine Faculty of Medicine and Health, UNSW Sydney Sydney New South Wales Australia; ^4^ The George Institute for Global Health UNSW Sydney Sydney New South Wales Australia

**Keywords:** activity‐based costing, cancer outcomes, health benefits, local control, overall survival, radiotherapy

## Abstract

**Introduction:**

Optimal radiotherapy (RT) use in cancer patients results in substantial 5‐year local control (LC) and overall survival (OS) benefits at the population level. This study aimed to estimate the average per capita cost of the first course of RT treatment and the associated cost per LC and OS outcomes, both overall and by cancer stage.

**Methods:**

Data on RT activities from 2017 to 2020 for lung, rectum, cervix, prostate, brain and head and neck (H&N) cancers were extracted from South‐Western Sydney Local Health District electronic oncology information system MOSAIQ (Elekta, version 2.63). Costs were assigned based on activity codes and adjusted for yearly inflation rates. The average cost per treatment course was calculated (average cost per activity × number of fractions). Costs per 5‐ and 1‐year LC and OS outcomes were estimated for all stages and for stages I–II and III.

**Results:**

A total of 106,174 RT activities were extracted. The average cost of an RT treatment course was highest for prostate cancer ($10,332) and lowest for lung cancer ($5598). The lowest costs per 5‐year outcome were observed for cervical cancers (LC: $15,780, OS: $28,370) and H&N cancers (LC: $17,500, OS: $29,750). The cost per 5‐year LC and OS outcome remained below $100,000 across all stages for each cancer type, except for prostate cancer, where the cost per OS outcome exceeded this level.

**Conclusion:**

This study demonstrates that the absolute costs associated with achieving 5‐year local control and overall survival outcomes with radiotherapy are comparatively low across several major cancer types. These findings highlight the efficiency of radiotherapy in delivering meaningful clinical outcomes and can help inform service planning, investment decisions and prioritisation of radiotherapy within cancer care strategies.

## 1 Introduction

In Australia, cancer accounts for 18% of total health burden, with approximately $9.7 billion Australian dollars spent on diagnosis and treatment of cancer [1]. Allocating economic resources effectively to improve health outcomes continues to be an evolving process.

Economic evaluation remains a priority for health services [2]. Various economic evaluation methods have been reported for costing of various health service components [3, 4]. Capturing all patient outcomes and associated costs is a time and resource‐intensive task that may not be feasible in all healthcare settings [5]. High‐quality data on current cancer care costs are needed to prioritise ongoing healthcare funding [6].

Evidence‐based models project that up to 48% of all cancer patients have indications for radiotherapy (RT) at least once during their illness [7]. If RT had been optimally used in all eligible patients, this would result in an 11% improvement in loco‐regional control (LC) and a 3% improvement in overall survival (OS) for up to 5 years at the population level for all cancers [8]. How this improvement in outcomes compares to the cost of providing RT at the population level has not been previously addressed. A combined activity‐based costing and relative value unit costing (ABC‐RVU) model that includes the costs of direct service provision activities as well as indirect costs of personnel and resource maintenance has been previously used for evaluating RT costs [9, 10, 11]. This approach was applied by New South Wales (NSW) Health and published by our group previously, where the costs of RT were analysed in relation to population level outcome gains in 5‐year LC and OS [12].

This study is a follow‐up study based on the cost assessment of the previous study by our group [12]. The data used in our previous study covered only 1 year of curative treatment and did not address costs by stage of cancer, treatment intent or include the costs of newer RT techniques.

In this study, the combined ABC‐RVU approach was used to estimate the cost of RT per fraction by stage for selected cancers using 3 years of RT activity data. This analytical study provides a detailed cost per outcome analysis of providing RT by stage at diagnosis for those selected cancers where optimal RT utilisation was estimated to be 50% or greater according to the optimal RT utilisation model [7]. These cancers include lung, prostate, cervix, rectum, brain and head and neck (H & N) cancers. Costs for breast cancer were also calculated and published separately [13].

The aims of this study were (1) to quantify the average per capita cost of the first course of RT treatment after diagnosis by cancer stage and (2) to estimate the cost per absolute LC and OS outcome based on RT alone for selected cancers in which RT is commonly used as part of curative multimodality therapy.

## 2 Methods

All RT activities (July 2017–June 2020) with curative and palliative intents for selected cancers were extracted from South‐Western Sydney Local Health District (SWSLHD) Cancer Service oncology information system MOSAIQ (Elekta, version 2.63). SWSLHD covers one of the largest geographic areas in NSW, with a population of over 1 million people [14]. The radiation oncology service within the district operates across two geographical sites with an additional two outreach clinics. It is equipped with seven linear accelerators and also offers brachytherapy (high‐dose rate only) and orthovoltage treatments. The average number of new courses of RT treatment per year is 2300 [15].

All external beam and brachytherapy techniques, including 3D‐conformal RT, intensity modulated RT (IMRT), volumetric modulated arc therapy (VMAT), Tomo Helical and deep‐inspiration breathhold (DIBH) were recorded for conventional fractionation and stereotactic ablative body radiotherapy (SABR). Demographic data on stage at diagnosis for cancer patients treated with RT during the study period were also extracted from the electronic database. Delivery costs of the total number of fractions of the first course of RT were assessed for various clinical scenarios (primary cancer or recurrent cancer previously untreated with RT), as published in our original study [12]. Pre‐treatment, treatment and post‐treatment RT activities for the first RT course associated with each tumour site were consolidated. For each patient, the cost of all activities, from the first radiation oncology consultation to the first follow‐up visit after treatment ended, was calculated. Individual RT activities included RT bookings, consultations, care coordination, pre‐simulation, simulation, planning, treatment and discharge for follow‐up. RT delivery and all associated RT‐related activities specific to the tumour site were included in the costing analyses.

The costs of individual activities and techniques such as VMAT/IMRT or conformal RT were recorded according to the cost codes previously assigned by our group. The initial cost allocation process published in Batumalai et al. study [12] involved assessment of costing of staff time input, energy input and RT equipment maintenance input, extracted from the departmental financial system. Expenses, including all direct and indirect labour, goods and services, repairs and maintenance and administration, were used to achieve a set of relative value units (RVU). RVUs represented the cost of each radiotherapy activity relative to the average cost of all activities and were used to achieve a weighted allocation. The final set of RVUs was used in conjunction with the activity extract from MOSAIQ to allocate radiotherapy costs across activities [12]. This approach ensured that the costing of all activities involved in the treatment course for individual patients was included. Costs related to other treatment services during the selected period, such as medical oncology‐related blood tests, were excluded. Other adjuvant or concurrent treatments for the patients, such as surgery, chemotherapy and related activities, were not included in our analyses.

The activity costs were quoted in Australian dollar ($AUD) and adjusted for inflation using Reserve Bank of Australia rates for the study period, as advised by a health economist [16]. This approach was selected to ensure consistency with standard economic evaluation methods. All radiotherapy activities (pre‐treatment, treatment and posttreatment) associated with each tumour site and the associated cost were consolidated together. This included one‐time activities (e.g., simulation, planning) and recurring activities (e.g., fraction), with each activity counted only once per occurrence. The cost per activity was determined by dividing the average total cost per patient by the average number of activities. As described in the methods presented in the original study, this cost per activity was assumed to be the cost per fraction to account for the cost of all activities involved in the treatment preparation. The average cost of the treatment course was thus calculated by multiplying the average cost per fraction by the average number of fractions delivered (average cost per fraction × average number of fractions).

**FIGURE 1:**
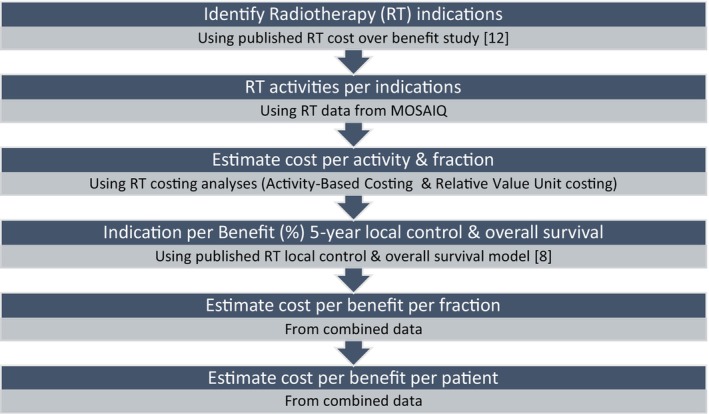
Methods flowchart.

Average costs of treatment courses were compared with outcomes (5‐year LC and OS) stratified by tumour stage. The overall cost per outcome ratio was determined based on previously developed models estimating the optimal benefit of RT alone in terms of LC and OS [8]. These models do not incorporate the effects of multimodality treatment approaches (e.g., surgery or systemic therapy). Cost per outcome ratios were estimated from the cost of total fractions over 5‐year LC and OS gain for all stages and for stages I–II and stage III separately. Costing for stage IV data were excluded from the cost‐outcome comparison as LC or OS outcomes of RT for stage IV were deemed zero [8]. Similar methods were applied for all selected cancer sites, stratified by stage at diagnosis and overall. An example of the calculations for the cost‐outcome (LC and OS) ratio for early‐stage (stage I–II) lung cancer is described in Table 1. The flowchart of the methods is illustrated in Figure 1.

**TABLE 1:** Example of calculation methods for cost per local control and overall survival outcomes for stage I–II lung cancer.

5‐year local control (LC)	19.7%
5‐year overall survival (OS)	13.20%
Average fractions per patient with stage I–II lung cancer	15
Average calculated costs per fraction	$392
Average calculated costs per treatment course (cost*fraction)	$5885
Fractions needed for 5‐year LC gain (fractions/LC benefit)	76
Fractions needed for 5‐year OS benefit (fractions/OS benefit)	114
Cost for 5‐year LC benefit ($392*76)	$29,871
Cost for 5‐year OS benefit ($392*114)	$44,580

## 3 Results

The study sample consisted of 1437 patients across six cancer sites (lung, rectum, cervix, prostate, brain and H&N) who were treated with RT over the defined study period. A total of 106,174 activities were recorded (Table 2). The proportion of RT activities delivered with curative intent ranged from 55.3% for lung cancer to 95.2% for brain cancer. The average number of fractions for all stages ranged from 18 for lung cancer to 29 for H&N cancer. The results for individual cancer sites are presented in Tables 3 and 4.

**TABLE 2:** Study population by radiotherapy delivery by tumour site.

	Lung	Rectum	Cervix	Prostate	Brain	Head and neck
**Activities (N)**	30,535	12,234	3184	18,403	9434	32,384
**Year**						
2017–2018	31.9%	43.1%	36.7%	35.4%	39.4%	35.0%
2018–2019	35.2%	28.8%	26.7%	37.5%	29.8%	35.8%
2019–2020	32.8%	28.1%	36.7%	27.0%	30.8%	29.2%
**Treatment intent**						
Curative	55.3%	75.0%	86.7%	92.3%	95.2%	90.0%
Palliative	44.7%	25.0%	13.3%	7.7%	4.8%	10.0%
**Stage**						
I	14.3%	11.3%	23.3%	1.6%		
II	7.9%	17.5%	20.0%	54.5%		
III	38.1%	47.5%	26.7%	19.3%		
IV	37.2%	18.8%	26.7%	17.0%		
Unknown	2.4%	5.0%	3.3%	7.5%		

Fraction # by stage	Median	Mean	Range	Median	Mean	Range	Median	Mean	Range	Median	Mean	Range	Median	Mean	Range	Median	Mean	Range
I	5	9	1–30	25	20	5–27	28	28	25–28	29	29	20–39						
II	20	18	1–33	25	19	5–27	29	27	13–28	20	27	5–39						
III	27	25	1–33	25	22	1–33	29	27	8–28	34	33	12–39						
IV	15	15	1–30	5	9	1–28	22	20	8–33	20	27	8–39	No	Stage		No	Stage	
All	20	18	1–33	25	19	1–33	28	25	8–33	34	28	5–39	30	23	3–33	33	29	

Dose (Gy) by stage	Median	Mean	Range	Median	Mean	Range	Median	Mean	Range	Median	Mean	Range	Median	Mean	Range	Median	Mean	Range
I	48	48	12–60	50	44	25–54	45	47	45–50	67	69	60–78						
II	55	48	6–66	50	43	25–54	45	47	26–55	64	66	26–78						
III	55	45	6–66	50	45	6–66	50	48	18–58	68	68	24–78	No	stage		No	stage	
IV	20	22	4–60	25	27	2–60	50	47	16–60	60	65	30–78						
All	36	37	4–66	50	41	2–66	45	47	16–60	68	66	24–78	60	51	6–66	66	61	

**TABLE 3:** Cost estimation by stage and treatment course for selected cancer sites.

	Average cost/fraction	Average fraction #	Cost AUD/RT course
**Lung**			
*All stages*	$311	18	$5598
Stage I–II	$392	15	$5885
Stage III	$295	25	$7363
Stage IV	$348	15	$5225
**Rectum**			
*All stages*	$327	19	$6208
Stage I–II	$337	20	$6750
Stage III	$330	22	$7262
Stage IV	$290	9	$2613
**Cervix**			
*All stages*	$320	25	$8000
Stage I–II	$338	27	$9114
Stage III	$293	27	$7899
Stage IV	$301	20	$6027
**Prostate**			
*All stages*	$377	27	$10,332
Stage I–II	$384	26	$9780
Stage III	$356	33	$11,812
Stage IV	$442	20	$8844
**Brain**			
*All stages*	$340	23	$7821
**Head and Neck**			
*All stages*	$277	29	$8032

**TABLE 4:** Cost over outcome calculation by stage (5‐year local control and overall survival).

	5‐yr LC%	Cost AUD/5‐yr LC	5‐yr OS%	Cost AUD/5‐yr OS
**Lung**				
*All stages*	12	$48,678	7	$86,123
Stage I–II	20	$29,871	13	$44,580
Stage III	13	$55,363	9	$82,733
Stage IV	—	—	—	—
**Rectum**				
*All stages*	22	$28,608	7	$92,657
Stage I–II	44	$15,340	22	$30,681
Stage III	22	$33,010	6	$121,037
Stage IV	—	—	—	—
**Cervix**				
*All stages*	51	$15,780	28	$28,370
Stage I–II	57	$16,130	42	$21,699
Stage III	77	$10,259	38	$20,787
Stage IV	—	—	—	—
**Prostate**				
*All stages*	43	$23,722	2	$601,412
Stage I–II	44	$22,490	2	$498,150
Stage III	46	$25,412	5	$292,875
Stage IV	—	—	—	—
**Brain**				
*All stages*	11	$69,214	10	$78,212
**Head & Neck**				
*All stages*	46	$17,500	27	$29,750

Average costs of first RT course were higher for prostate ($10,332) and H&N ($8032) cancers and lower for lung ($5598) and rectal ($6208) cancers. The average costs over 5‐year LC gain for all stages were below $25,000 for cancers with higher LC outcomes, such as cervical, H&N and prostate cancers ($15,780–$23,722 per 5‐year LC). Similarly, the costs for 5‐year OS gain were lowest for cervical and H&N cancers, while prostate cancer had the highest cost due to minimal OS benefit from RT ($601,412) (Table 4).

When comparing tumour sites, cancers that required fewer than 20 fractions had lower costs compared to those requiring more fractions. Stage III prostate cancer had the highest average number of fractions, and its cost estimation was the highest at $11,812 per treatment course. Stage IV cancers with palliative intent required fewer fractions and hence had lower costs. For stage IV rectal cancer, the average number of fractions was nine, and the average cost was only $2613 per treatment course.

**FIGURE 2:**
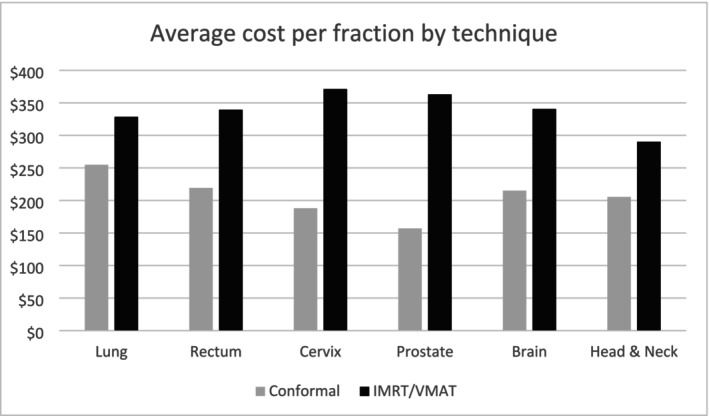
Cost by radiotherapy techniques and tumour site.

IMRT or VMAT technique was more frequently used for brain, H&N, prostate and rectal cancers. For brain cancer, all but one patient received RT using VMAT or IMRT. The majority of H&N cancer‐related RT activities were delivered using IMRT, Tomo Helical or VMAT techniques. For rectal cancer, over 50% of RT activities involved VMAT or IMRT. Average costs for more sophisticated RT techniques such as VMAT or IMRT were higher compared to 3D‐conformal techniques. The average cost of treatment using VMAT or IMRT for all sites was over $320 per fraction, with the lowest cost for H&N cancers ($290) (Figure 2).

For cancers with available stage data, costs for LC or OS outcomes were considered by stage. The cost of treating early (stage I–II) versus late stage (stage III) cancers depended on the number of fractions in the treatment course as well as the 5‐year benefits of LC and OS. Costs for LC and OS for earlier stages were comparatively lower where the average number of fractions was less than those for higher stage cancers as in lung and rectal cancers (Table 4). Stage III cervical cancer had the lowest cost for both LC ($10,259) and OS ($20,787) as RT benefit for LC (77%) and OS (38%) were one of the highest for advanced stage cervical cancers. For rectal cancers, the RT costs for stage I–II versus stage III were half for LC outcomes ($15,340 vs. $33,010) and one‐fourth for OS outcome ($30,681 vs. $121,037). For prostate cancer, the cost per LC outcome for stage I–II compared to stage III was comparable to other sites ($22,490 vs. $25,412). The OS outcome from RT for prostate cancer for all stages was the lowest among all selected sites (2.3% to 4.5%), resulting in the highest cost per outcome between stages ($498,150 vs. $292,875). Cost estimations are presented in Table 4.

Sixty‐four % (*n* = 19) of all cervical cancer patients also received brachytherapy, with 47% in stage I–II and 21% in stage III, while only three patients in stage IV received brachytherapy. The average cost of brachytherapy was estimated at $338 per activity.

## 4 Discussion

This study presents an analysis of RT treatment delivery costs with a comprehensive comparison with traditional outcomes measures (5‐year LC and OS) based on the benefits of RT alone. This provides a descriptive, population‐level view of cost per outcome using routine health service data. We found that cancers with higher LC and OS benefits with RT had the lowest absolute costs per benefit. With the exception of prostate cancer, the estimated costs across all stages were generally lower than published monetary values of other cancer treatment services, which often exceed $100,000 per year [17]. The high cost‐outcome ratio for prostate cancer reflects the small survival gain with RT and the protracted fractionation schedules. However, this is likely to change in the future due to the increasing use of SABR in prostate cancer, which typically involves five fractions [18], and emerging evidence suggesting two fractions [19].

The most significant costing component of RT is the technical costs associated with treatment delivery to individual patients. Limited literature on initial technical costs of providing RT has favoured detailed activity‐based costing over Medicare reimbursement costing used previously [20]. Our approach, incorporating costs of building/infrastructure, equipment and various professionals (e.g., radiation oncologists, radiation therapists, medical physicists, nurses, receptionists, psychologists, etc.) has been a major strength in this study.

Radiotherapy treatment costing studies for specific treatment options have been reported globally. Few studies have reported different costing valuations of specific RT therapies for specific cancers. A recent systematic overview of costs and resources for early‐stage non‐small cell lung cancer highlighted the need for more comprehensive cost evaluations to guide health funding organisations [21]. There are also few studies that explored the costs of preventing recurrence and providing palliative treatments for bone metastasis or pain management [22, 23, 24].

Our study has limitations. The cost per outcome figures presented here are absolute, not incremental. They describe the cost required to achieve one unit of benefit from RT alone, but do not constitute cost‐effectiveness ratios in the formal health economic sense. Cost‐effectiveness analyses require comparative or incremental evaluations. Therefore, while our findings offer insight into the magnitude of investment per clinical benefit, they should not be interpreted as evidence of cost‐effectiveness.

Our study has not had the scope of measuring RT delivery cost from the patient perspective, including palliative RT, which is reported as a burden for individual cancer patients who are offered RT [25]. Therefore, our estimates should not be used, alone, as a decision rule for a measure of affordability. As mentioned in a policy review literature, a fixed effectiveness threshold level may not be the only measure for funding decisions; confidence in the clinical data has been just as important as estimated cost–effectiveness ratios, as described by the reviewers of the Australian Pharmaceutical Benefits (PBS) program [26]. Our projected cost‐outcome ratios should be interpreted as indicators of the absolute cost associated with RT‐derived benefits in routine practice.

Our model‐based benefit projections may have influenced the cost‐outcome ratios negatively in certain scenarios. For example, in prostate cancer, especially early‐stage disease, higher costs were estimated relative to survival outcomes, which may suggest that RT offers lower value for money in these cases. The cost‐outcome ratio calculations relied on model‐based projections of LC and OS outcomes derived from RT alone [8], without consideration of potential survival or disease control gains from surgery or systemic therapy when delivered as part of a multimodality approach. This is particularly relevant for tumour types such as prostate and pancreatic cancer, where combination treatment is common and typically improves outcomes. Conversely, RT for cervical cancer, where evidence‐based outcomes are among the highest, provided the lowest cost for outcome valuation. Literature from low‐income countries in Africa and Asia [27, 28, 29] has reported higher prevalence of cervical cancers and a high demand for cancer services for these cancers. Poor access to RT reported in lower‐ and middle‐income countries (LMIC) highlights the urgent need for establishment of RT services to meet this demand [30]. The lower costs of RT for estimated survival outcomes in our study would encourage the local health services and funding organisations to invest in RT facilities in these countries. Although this study did not include a formal sensitivity analysis of the costing estimates, the outcome models on which the LC and OS estimates are based incorporated sensitivity analyses in their original development. Future studies may benefit from including sensitivity analyses of cost assumptions to further support the robustness of cost‐outcome estimates.

The data are based on one large radiotherapy service in metropolitan Sydney and may not represent the case mix in other areas of Australia or other countries. Additionally, the cost‐outcome ratio calculated in our study is based on value assigned to cost of fractions, including the treatment preparations costs such as contrast, follow‐up, pre‐planning imaging, which may vary between different cancers. We selected RT and related activities excluding any activities before the first appointment date with the radiation oncologist and any activities after the treatment finish date or first follow‐up date if available on the record. Therefore, costs may have been overestimated for cancers with more complex treatment preparation compared to those with less complex preparation. Average costs per fraction for more complex treatments are higher than those for less complex 3D‐conformal techniques. Cost analyses of a larger number of activities from a diverse patient base over a three‐year period may have minimised the overappraisal to some extent. It may have been more accurate to calculate the costs incurred in the treatment preparation stage separately [11]; however, this approach would have been more time‐consuming and was beyond the scope of this study.

Cost analyses presented in this study were set in a high‐income country and may not be representative of costs in LMIC. Costs will vary between regions due to differing capital and human resource costs. The proportion of patients with cancer in LMIC ideally would benefit more from RT due to cancer types and stages at diagnosis [28]. In low‐income countries, the establishment of equipment and personnel costs is lower compared to high‐income countries; therefore, our findings are not directly generalisable to those country settings. Nevertheless, our comprehensive costing evaluation over long‐term health outcomes with evaluation of a sizeable number of RT activities indicates that government spending on RT services would be well utilised.

## 5 Conclusion

The economic burden of premature disability and deaths from cancer is high. This study offers a descriptive assessment of absolute costs per outcome (LC and OS) achieved by RT alone across selected cancer types. While these metrics do not represent formal cost‐effectiveness ratios, they provide useful insights into the relative cost burden and potential return on investment from RT in routine clinical settings. Monetary value estimates of costs relative to population‐level long‐term outcomes such as recurrence prevention and OS can guide the allocation of spending for RT services and address any service shortfalls for specific cancer populations. Further research using detailed economic evaluation methods is recommended, especially in lower‐income countries that require substantial prioritisation to optimise the use of limited funding.

## Ethics Statement

In compliance with South‐Western Sydney Local Health District’s institutional ethics policy this project was exempt from requiring research ethics approval.

## Conflicts of Interest

Prof Shalini Vinod is a Member of the Editorial Board for JMIRO but was not involved in editorial handling or reviewer selection for this manuscript.

## Data Availability

The data that support the findings of this study are available on request from the corresponding author. The data are not publicly available due to privacy or ethical restrictions.
